# Supplementary material for: Andrographolide induces protective autophagy and targeting DJ-1 triggers reactive oxygen species-induced cell death in pancreatic cancer
Source: PeerJ. 2024 Jun 28;12:e17619. doi: 10.7717/peerj.17619 (PMC11216212; doi:10.7717/peerj.17619)
Supplement: Supplemental Information 1 [file peerj-12-17619-s001.zip › English-language codebook.docx]

- 复孔: Duplicate well

- 原始数据: Raw data

- 数据处理: Data processing

- 空白孔平均值: Blank well mean

- 存活率: Survival rate

- 标准品浓度: Standard concentration

- 标准管: Standard tube

- X值: X value

- y值: Y value

- 浓度拟合曲线: Concentration fitting curve

- 样本编号: Sample number

- 测定管: Measurement tube

- 对照管: Control tube

- 样本蛋白质浓度: Sample protein concentration

- LDH活力: LDH activity

- 测量日期: Measurement date

- 接种日期: Inoculation date

- 肿瘤体积: Tumor volume

- 备注: Note

- 数据1:data 1

- 标签: Tag

- 面积: Area

- 灰度值（平均值）: Gray value (average)

- 灰度绝对值: Gray absolute value

- 总灰度值: Total gray value

- 背景: Background

- 内参GAPDH: Internal control GAPDH

- 分组: Grouping

- 基因名: Gene name

- 双向引物序列: Bidirectional primer sequence
