# Supplementary figures and images for: Andrographolide induces protective autophagy and targeting DJ-1 triggers reactive oxygen species-induced cell death in pancreatic cancer
Source: PeerJ. 2024 Jun 28;12:e17619. doi: 10.7717/peerj.17619 (PMC11216212; doi:10.7717/peerj.17619)

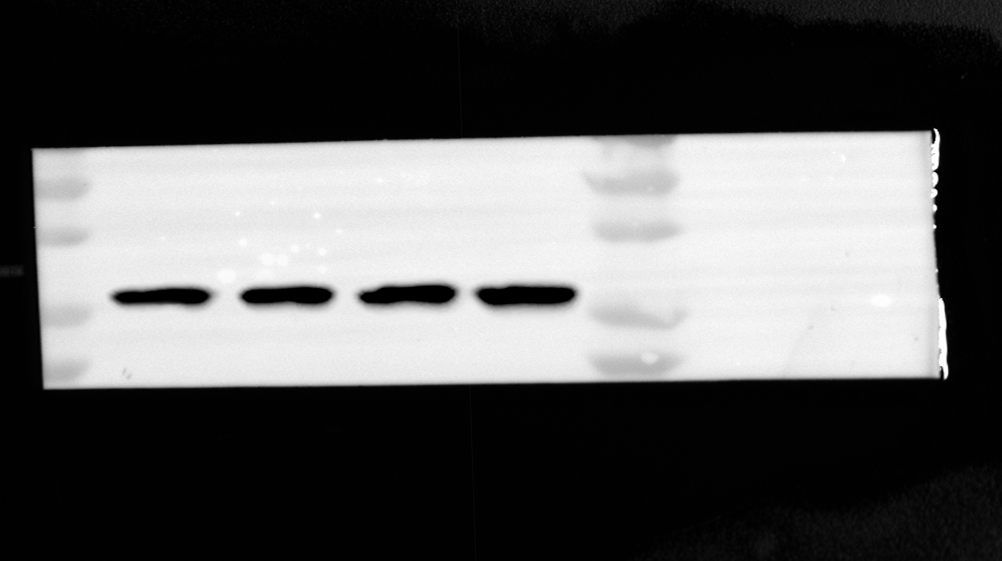

Supplement: Supplemental Information 2 [file peerj-12-17619-s002.zip › figure 4/GAPDH.tif]

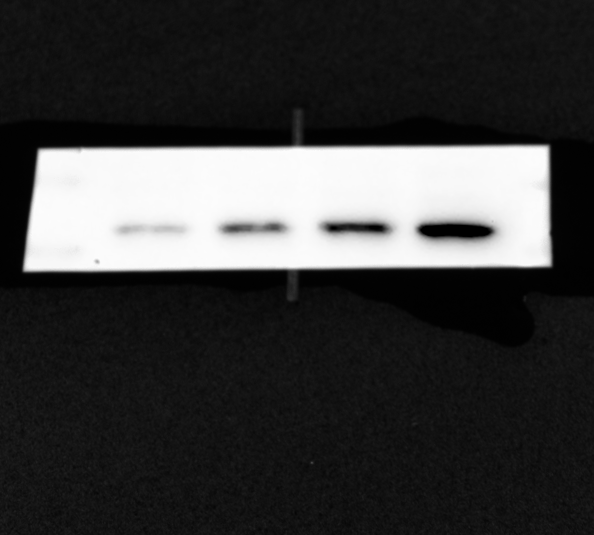

Supplement: Supplemental Information 2 [file peerj-12-17619-s002.zip › figure 4/cleaved-caspase 3.tif]

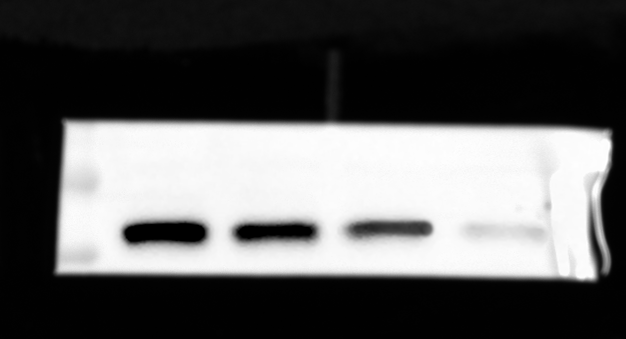

Supplement: Supplemental Information 2 [file peerj-12-17619-s002.zip › figure 5a/DJ-1.tif]

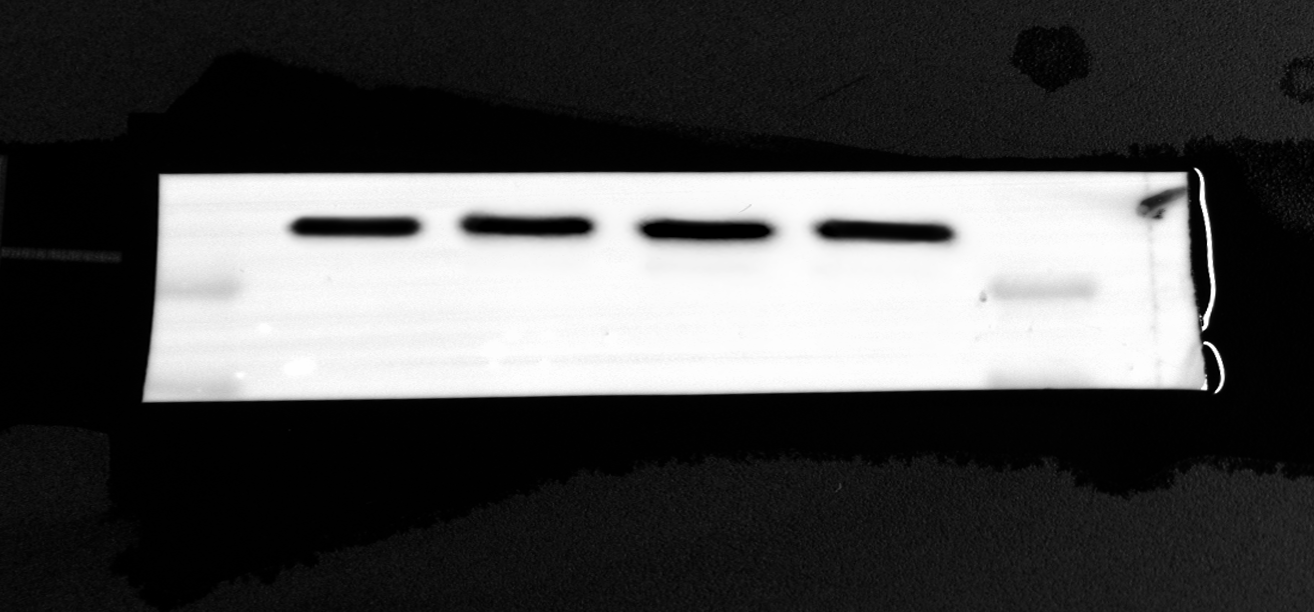

Supplement: Supplemental Information 2 [file peerj-12-17619-s002.zip › figure 5a/GADPH.tif]

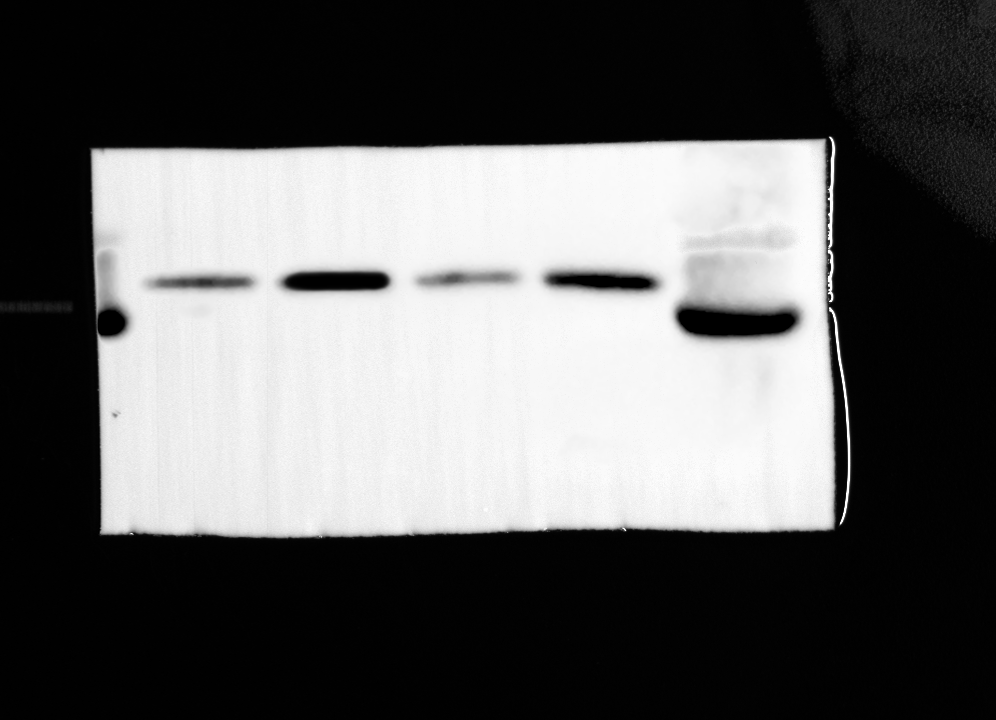

Supplement: Supplemental Information 2 [file peerj-12-17619-s002.zip › figure 5i/Cleaved caspase 3.tif]

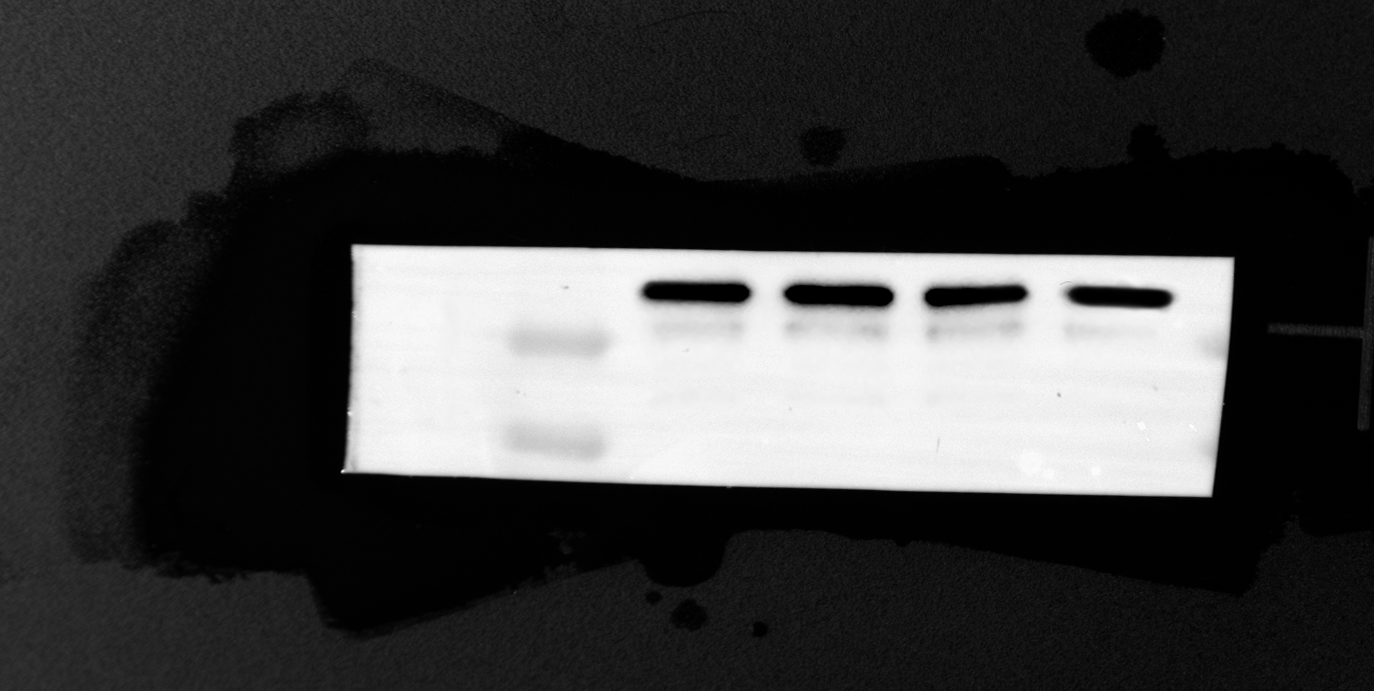

Supplement: Supplemental Information 2 [file peerj-12-17619-s002.zip › figure 5i/GADPH.tif]

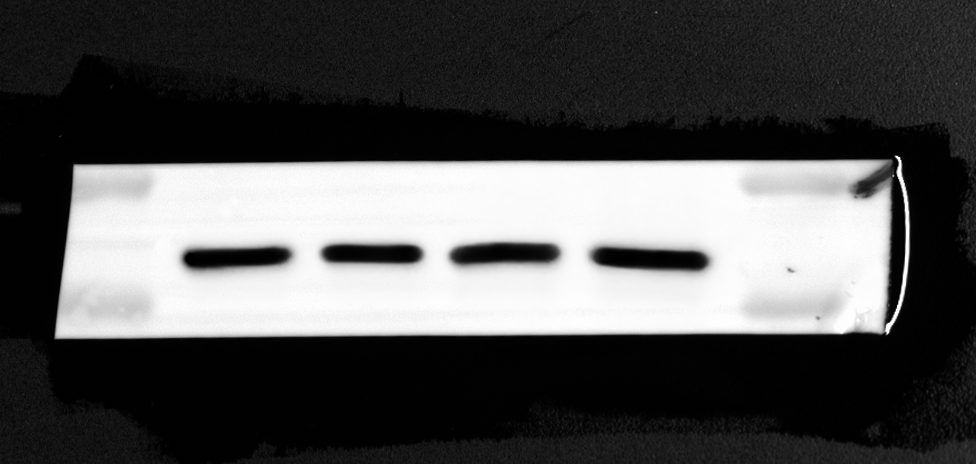

Supplement: Supplemental Information 2 [file peerj-12-17619-s002.zip › figure 6b/GADPH.tif]

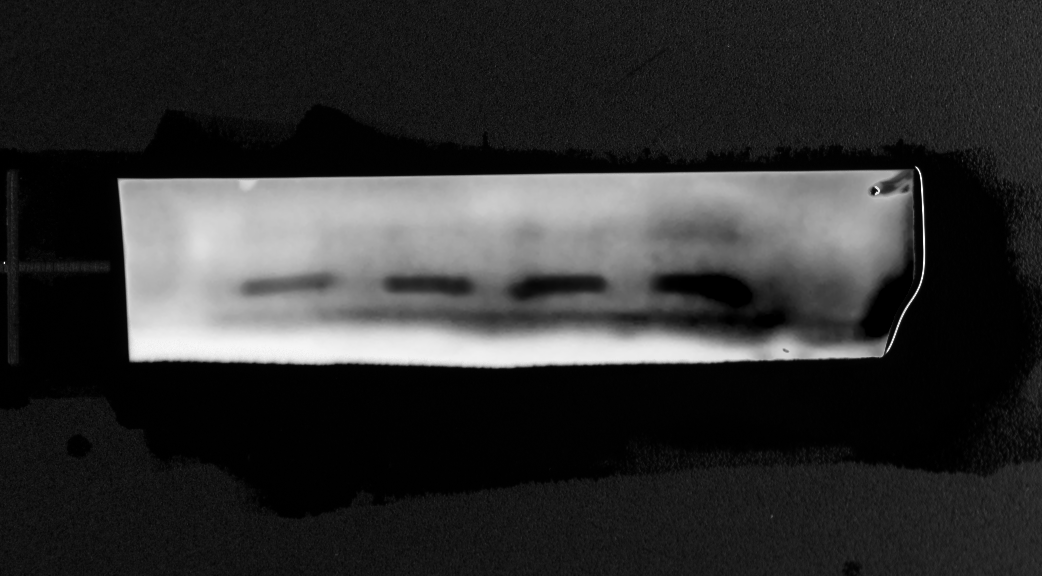

Supplement: Supplemental Information 2 [file peerj-12-17619-s002.zip › figure 6b/LC3.tif]

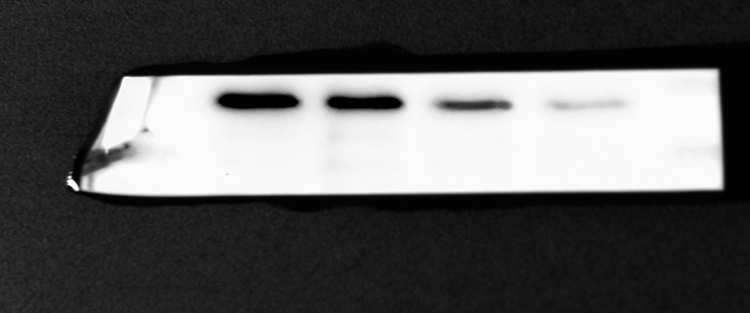

Supplement: Supplemental Information 2 [file peerj-12-17619-s002.zip › figure 6b/p62.tif]

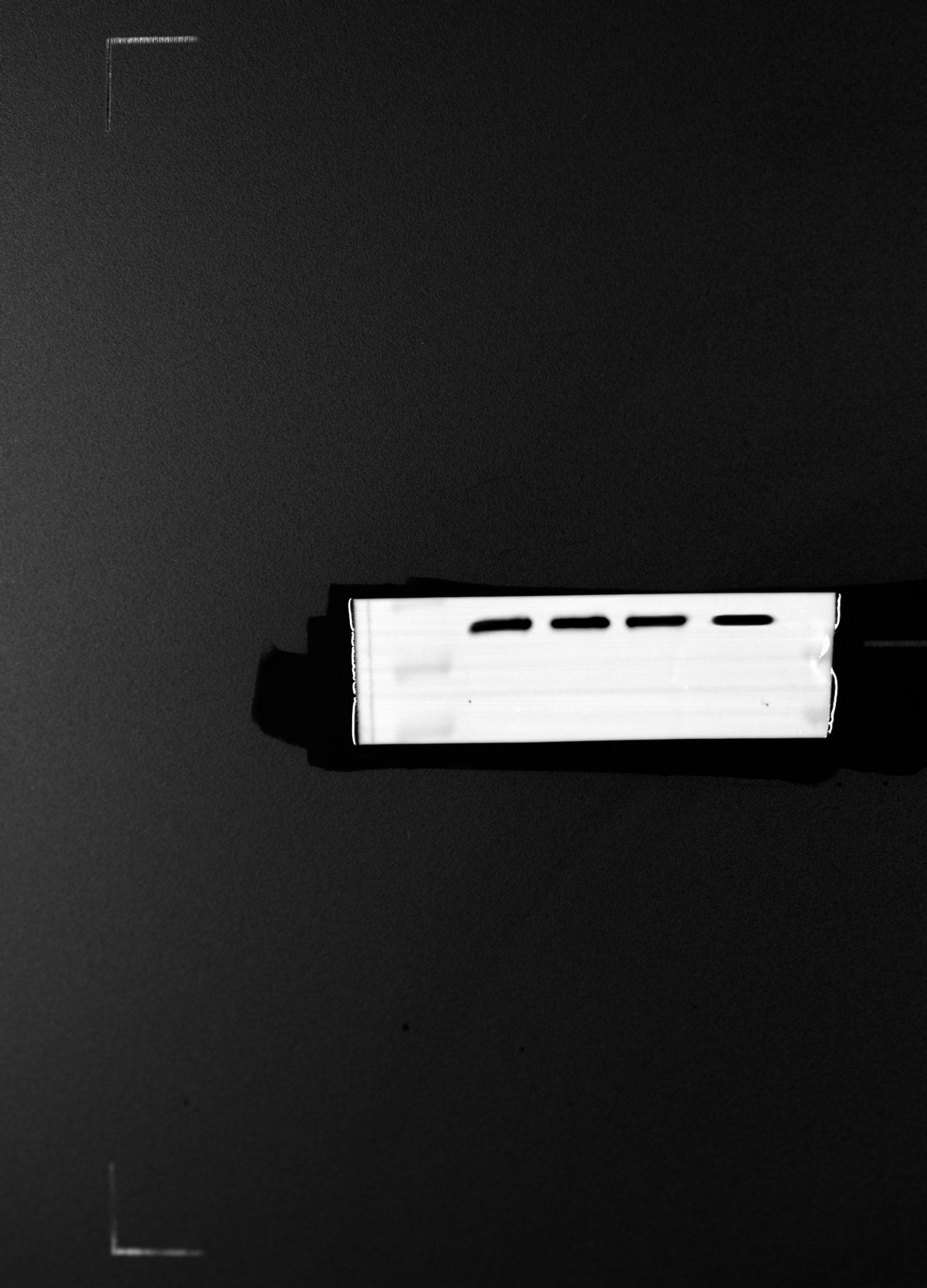

Supplement: Supplemental Information 2 [file peerj-12-17619-s002.zip › figure 6j/GADPH.tif]

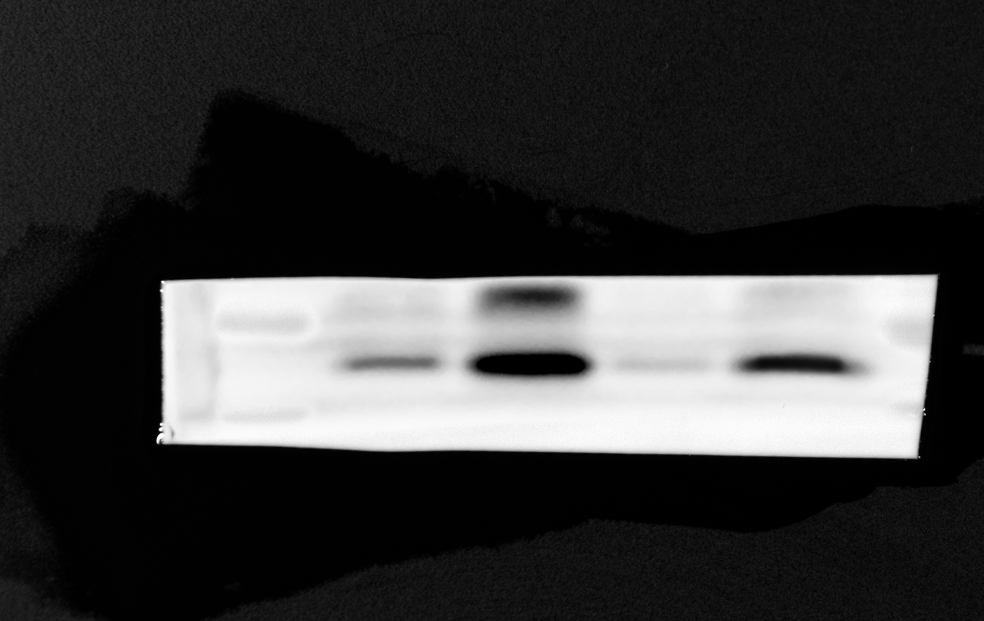

Supplement: Supplemental Information 2 [file peerj-12-17619-s002.zip › figure 6j/LC3.tif]

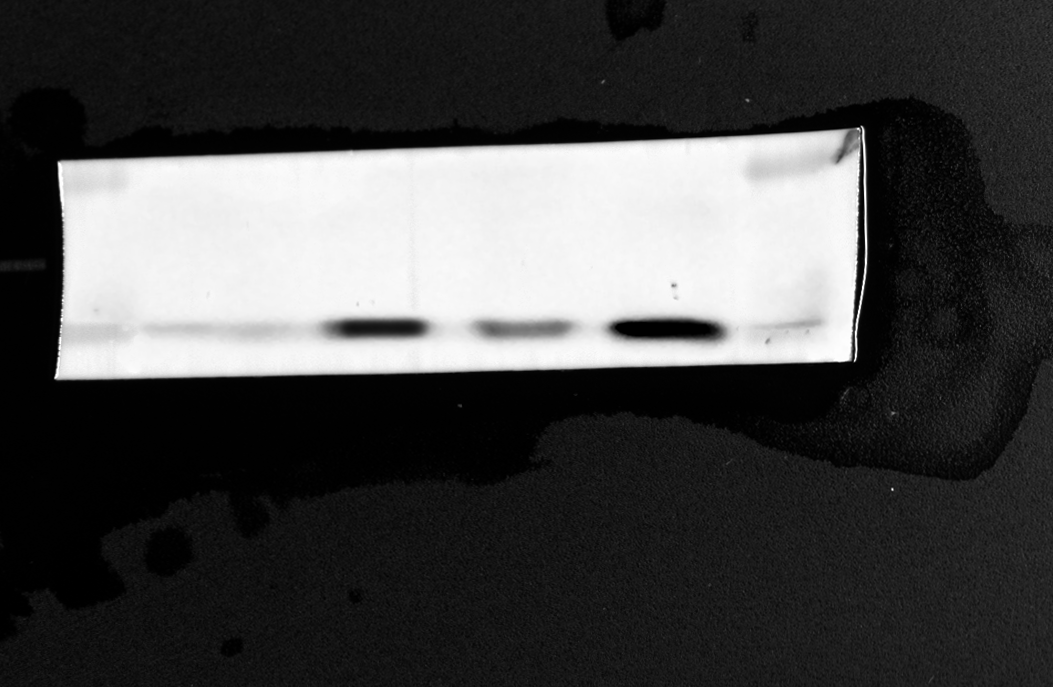

Supplement: Supplemental Information 2 [file peerj-12-17619-s002.zip › figure 6j/cleaved-caspase 3.tif]

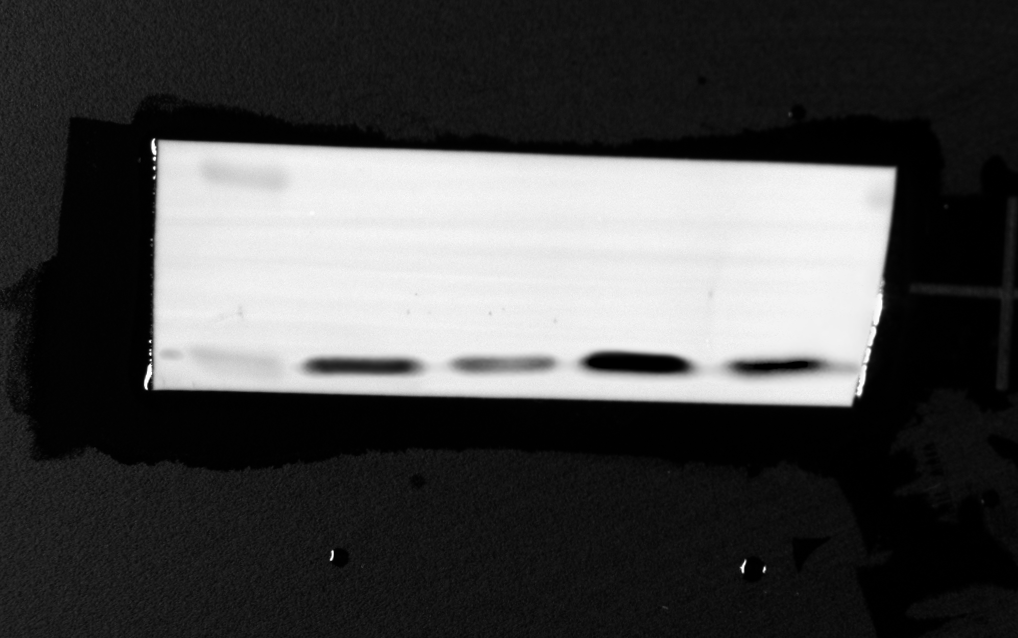

Supplement: Supplemental Information 2 [file peerj-12-17619-s002.zip › figure 6j/p62.tif]
