# Supplementary material for: Andrographolide induces protective autophagy and targeting DJ-1 triggers reactive oxygen species-induced cell death in pancreatic cancer
Source: PeerJ. 2024 Jun 28;12:e17619. doi: 10.7717/peerj.17619 (PMC11216212; doi:10.7717/peerj.17619)
Supplement: Supplemental Information 5 [file peerj-12-17619-s005.pdf]

Table 1. MIQE checklist for authors, reviewers, and editors.[a]

Item to check	Importance	Item to check	Importance
Experimental design		qPCR oligonucleotides	
Definition of experimental and control groups	line 549	Primer sequences	Table 1
Number within each group	line 189	RTPrimerDB identification number	×
Assay carried out by the core or investigator's laboratory?	line 389	Probe sequences	×
Acknowledgment of authors' contributions	line 389	Location and identity of any modifications	×
Sample		Manufacturer of oligonucleotides	×
Description	line 183	Purification method	line 184
Volume/mass of sample processed	√	qPCR protocol	
Microdissection or macrodissection	×	Complete reaction conditions	line186
Processing procedure	line 183	Reaction volume and amount of cDNA/DNA	line 185
If frozen, how and how quickly?	√	Primer, (probe), Mg^{2+}, and dNTP concentrations	line 186
If fixed, with what and how quickly?	×	Polymerase identity and concentration	×
Sample storage conditions and duration (especially for FFPE[b] samples)	×	Buffer/kit identity and manufacturer	line 188
Nucleic acid extraction		Exact chemical composition of the buffer	√
Procedure and/or instrumentation	line 183	Additives (SYBR Green I, DMSO, and so forth)	line 188
Name of kit and details of any modifications	line 183	Manufacturer of plates/tubes and catalog number	line 188
Source of additional reagents used	×	Complete thermocycling parameters	√
Details of DNase or RNase treatment	line 183	Reaction setup (manual/robotic)	×
Contamination assessment (DNA or RNA)	line 183	Manufacturer of qPCR instrument	line 188
Nucleic acid quantification	√	qPCR validation	
Instrument and method	line 183	Evidence of optimization (from gradients)	×
Purity (A_{260}/A_{280})	√	Specificity (gel, sequence, melt, or digest)	×
Yield	√	For SYBR Green I, C_q of the NTC	original data
RNA integrity: method/instrument	√	Calibration curves with slope and *y* intercept	√
RIN/RQI or C_q of 3′ and 5′ transcripts	original data	PCR efficiency calculated from slope	√
Electrophoresis traces	×	CIs for PCR efficiency or SE	×
Inhibition testing (C_q dilutions, spike, or other)	×	r^2 of calibration curve	√
Reverse transcription		Linear dynamic range	√
Complete reaction conditions	√	C_q variation at LOD	×
Amount of RNA and reaction volume	√	CIs throughout range	×
Priming oligonucleotide (if using GSP) and concentration	√	Evidence for LOD	×
Reverse transcriptase and concentration	line 184	If multiplex, efficiency and LOD of each assay	√
Temperature and time	√	Data analysis	
Manufacturer of reagents and catalogue numbers	√	qPCR analysis program (source, version)	line 188
C_qs with and without reverse transcription	original data	Method of C_q determination	line 188
Storage conditions of cDNA	×	Outlier identification and disposition	√
qPCR target information		Results for NTCs	×
Gene symbol	table 1	Justification of number and choice of reference genes	√
Sequence accession number	×	Description of normalization method	line 190
Location of amplicon	√	Number and concordance of biological replicates	×
Amplicon length	√	Number and stage (reverse transcription or qPCR) of technical replicates	line 182
In silico specificity screen (BLAST, and so on)	√	Repeatability (intraassay variation)	line 190
Pseudogenes, retropseudogenes, or other homologs?	×	Reproducibility (interassay variation, CV)	√
Sequence alignment	×	Power analysis	×
Secondary structure analysis of amplicon	×	Statistical methods for results significance	line 190
Location of each primer by exon or intron (if applicable)	×	Software (source, version)	line 230
What splice variants are targeted?	×	C_q or raw data submission with RDML	original data

[a] All essential information (E) must be submitted with the manuscript. Desirable information (D) should be submitted if available. If primers are from RTPrimerDB, information on qPCR target, oligonucleotides, protocols, and validation is available from that source.

[b] FFPE, formalin-fixed, paraffin-embedded; RIN, RNA integrity number; RQI, RNA quality indicator; GSP, gene-specific priming; dNTP, deoxynucleoside triphosphate.

[c] Assessing the absence of DNA with a no–reverse transcription assay is essential when first extracting RNA. Once the sample has been validated as DNA free, inclusion of a no–reverse transcription control is desirable but no longer essential.

[d] Disclosure of the probe sequence is highly desirable and strongly encouraged; however, because not all vendors of commercial predesigned assays provide this information, it cannot be an essential requirement. Use of such assays is discouraged.
